# Relationships between Physical Activity and Quality of Life in Pregnant Women in the Second and Third Trimester

**DOI:** 10.3390/ijerph15122745

**Published:** 2018-12-05

**Authors:** Justyna Krzepota, Dorota Sadowska, Elżbieta Biernat

**Affiliations:** 1 Department of Physical Culture and Health Promotion, University of Szczecin, al. Piastów 40b, blok 6, 71-065 Szczecin, Poland; 2 Department of Physiology, Institute of Sport—National Research Institute, ul. Trylogii 2/16, 01-982 Warsaw, Poland; sadowska.dorota@hotmail.com; 3 Department of Tourism, Collegium of World Economy, Warsaw School of Economics, al. Niepodległości 162, 02-554 Warsaw, Poland; elzbieta.biernat@sgh.waw.pl

**Keywords:** physical activity in pregnancy, PPAQ, quality of life, WHOQoL-Bref, pregnant women

## Abstract

Background: The problem of an inadequate level of physical activity (PA) is important in the context of its relationship with the quality of life (QoL) of pregnant women. The aim of this study was to analyze the relationships between PA and QoL among pregnant women. Methods: The study analyzed 346 questionnaires filled in by pregnant women (157 in the second trimester and 189 in the third). The tool used for assessment of PA was the Pregnancy Physical Activity Questionnaire-Polish version (PPAQ-PL). The quality of life (QoL) was assessed by the World Health Organization Quality of Life Questionnaire-short form (WHOQoL-Bref). The results obtained from the PPAQ-PL and WHOQoL-Bref questionnaires for women in the second and third trimesters of pregnancy and intergroup differences were analyzed. Results: There was a significant correlation in the group of women in the second trimester of pregnancy between quality of life in the *physical health domain* and the intensity and type of physical activity. The women who rated their quality of life higher in this domain declared higher energy expenditures (EE) associated with *vigorous activity* (*R* = 0.159, *p* ≤ 0.05), as well as with *occupational activity* (*R* = 0.166; *p* ≤ 0.05) and *sport*/*exercise* activity (*R* = 0.187; *p* ≤ 0.05). In women in the third trimester, higher EE related to *sport*/*exercise activity* coincided with higher assessments of the *overall quality of life* (*R* = 0.149, *p* ≤ 0.05) and *general health* (*R* = 0.170, *p* ≤ 0.05). In the case of the *psychological domain* (*R* = 0.161, *p* ≤ 0.05) and *social relationship domain* (*R* = 0.188; *p* ≤ 0.05) of QoL, positive correlations occurred with EE related to *vigorous activity*. In contrast, high assessment of *physical health domain* coincided with higher EE related to *occupational activity* (*R* = 0.174; *p* ≤ 0.05). Conclusions: Our study makes an important contribution to knowledge concerning the correlations between PA and QoL in pregnancy. The results suggest the need for improvement in prenatal care and promotion of PA programs for pregnant women.

## 1. Introduction

The prevalence of an insufficient level of physical activity (PA) in pregnant women has been demonstrated in studies using representative samples in different countries [1,2,3,4]. Despite the well-documented benefits of involvement in PA in this period of life [5,6,7], it is emphasized that pregnancy continues to be one of the causes of a substantial reduction in PA [8,9,10].

Over the past two decades, most studies which have focused on these problems have estimated that most pregnant women do not participate in recommended PA. Clark and Gross [11] demonstrated that 39% of women who reported participating in some forms of weekly exercise before pregnancy did not report pursuing any similar activities during pregnancy. Similarly, Fell et al. [12], in a comparative study of women’s levels of PA during early pregnancy and during the year before pregnancy, observed that most women reduced their PA levels during the first 20 weeks of pregnancy compared with their level of activity during the year prior to pregnancy.

According to the data presented by Evenson et al. [1], only 15.8% of pregnant women in the USA reported being active, in accordance with the recommendations. In a cohort study conducted in Brazil, Domingues and Barros [2] estimated that only 4.3% of women were active during the whole pregnancy. Furthermore, [13] found that only 14.6% of women in mid-pregnancy in Norway were involved in the exercise ≥3 times a week, >20 min at moderate intensity. According to Santo et al. [4], merely 9% of 1584 pregnant women met the American College of Obstetrics and Gynecology ACOG guidelines. Furthermore, nearly half of the women reported PA < 1 day/week during the third trimester. Similarly, Haakstad et al. [3] found that only 11% of pregnant women followed ACOG guidelines regarding PA.

The problem of inadequate levels of PA is particularly important in the context of the relationship of PA to the quality of life (QoL) of pregnant women. Recently published studies have provided important information on changes that occur in health-related QOL (HRQoL), both during pregnancy and [14] in the perinatal period [15]. A problem that remains to be discussed is the explanation of the relationships between PA and the quality of life of pregnant women [16,17,18,19,20]. No unambiguous findings have been published to date in this area, since interpretation is difficult due to the use of different measurement tools, both for the evaluation of PA during pregnancy and the quality of life. For example, some researchers have used the results obtained with the Global Physical Activity Questionnaire (GPAQ) and 36-Item Short-Form Health Survey (SF-36) [20], while others have used the Pregnancy Physical Activity Questionnaire (PPAQ) and the abridged World Health Organization Quality of Life (WHOQoL-Bref) [16,18]. In addition to these tools, other questionnaires have been popular and are frequently used in the assessment of the quality of life, with an extensive review and discussion of their use being presented by Mogos et al. [21].

Despite many analyses, the problem of correlations between PA and the quality of life of pregnant women remains to be solved and still raises many questions that need to be addressed. To the best of our knowledge, the few studies that have used the PPAQ and WHOOoL-Bref tools [16,18] have failed to analyze the Polish population. Therefore, the aim of this study was to obtain information about the relationships between PA and QoL among pregnant women in Poland. The QoL was evaluated by a reliable questionnaire used in surveys of pregnant women (WHOQoL-Bref) [16,22,23,24], whereas PA was measured using the increasingly popular PPAQ questionnaire [25,26,27,28,29,30,31,32,33,34]. Furthermore, we hope that the choice of the above measurement tools will allow for replication of the study results and comparison with future research conducted on a wider scale.

## 2. Material and Methods

The study analyzed 346 questionnaires filled in correctly by pregnant women (157 in the second trimester and 189 in the third). The data were directly distributed among pregnant women who participated in activities for pregnant women organized within antenatal classes or in fitness clubs in Szczecin and Warsaw, Poland. The survey was anonymous and conducted only in places where consent was obtained. The respondents consisted only of volunteers. In each case, the survey supervisor presented the purpose and scope of the research to the respondents and instructed them on how to complete the questionnaires. The method “pen and paper” was used in the study. The respondents were given unlimited time to fill in the questionnaires.

The analysis excluded three questionnaires in which the first trimester of pregnancy was declared. Furthermore, the questionnaires which were not filled in completely or were filled in incorrectly were rejected (contrary to the instructions). 400 questionnaires were distributed to women, 346 of which were fully and properly completed. 21 questionnaires were incompletely or incorrectly filled in, and 29 questionnaires were not returned. The project was approved by a local Bioethics Committee.

The age of the respondents was 30.4 ± 3.6 years. Over 90% of study participants were college graduates (91.9%), 8.1% graduated from high school, 81.5% were married, 17.3% were single, while 1.2% were divorced. Furthermore, 88.2% were childless, 10.1% had 1 child and 1.7% had 2 or more children.

The tool used for assessment of PA was the Pregnancy Physical Activity Questionnaire-Polish version (PPAQ-PL) [32,33,34]. The original version of PPAQ was designed by Chasan-Taber et al. [35]. The questionnaire is used by pregnant women to self-assess their PA in the current trimester. In PPAQ-PL, the respondents were asked to report the time spent on participation in 32 types of activities grouped under the following categories: *household*/*caregiving* (12 activities), *occupational* (5 activities), *sports*/*exercise* (9 activities = 7 questions + two open questions, allowing the respondent to add any activities not previously listed), *transportation* (3 activities), and *inactivity* (3 activities). The questionnaire measures energy expenditure related to *total activity* and *total activity of light intensity and above* expressed in Metabolic Equivalent of Task (MET) units (MET-h∙week^−1^). Based on the energy expenditure, each of these activities was additionally classified according to intensity: (a) *sedentary activity* [<1.5 METs], (b) *light intensity activity* [1.5–<3.0 METs], (c) *moderate intensity activity* [3.0–6.0 METs], (d) *vigorous-intensity activity* [>6.0 METs]. The MET values were assigned according to the values presented in the questionnaire instruction and the Compendium of Physical Activities [36].

The methodological basis for the assessment of the quality of life (QoL) was provided by the abridged World Health Organization Quality of Life Questionnaire (WHOQoL-Bref), a Polish version provided by Wołowicka and Jaracz [37]. The WHOQoL-Bref questionnaire assesses self-reported QoL and general health of respondents. The WHOQoL-Bref questionnaire consists of 26 questions. The first two questions were analyzed separately. They concerned self-assessed *overall quality of life* and *general health* of the respondents. The remaining 24 questions assessed four domains of the QoL (*physical health domain*: 7 questions, *psychological domain*: 6 questions, *social relationships domain*: 3 questions, and *environmental domain*: 8 questions). The respondents were asked to mark their answers using a five-level rating scale (from 1 to 5 points, in a positive direction: the higher the number of points, the better quality of life). The QoL in the domains was expressed as mean values, calculated according to the key and guidelines provided by the authors [37].

STATISTICA 12.5 software was used for statistical analysis. The significance of the analyzed variables in women in the second and third trimesters of pregnancy was evaluated by means of the Mann-Whitney U-test. Correlations between the variables were analyzed using Spearman’s rank correlation test, with correlation coefficients calculated for each pair of variables. The level of statistical significance was set at *p* ≤ 0.05.

## 3. Results

The results obtained from the PPAQ-PL and WHOQoL-Bref questionnaires for the women in the second and third trimesters of pregnancy and intergroup differences (between the second and third trimesters) are presented in Table 1 and Table 2.

No statistically significant differences in the declared values of total energy expenditure (*total activity* and *total activity of light intensity and above*) were found among the women surveyed (Table 1). However, it was shown that the PA intensity differed significantly (*p* ≤ 0.05) depending on the trimester of pregnancy. This concerns in particular *sedentary activity* (group of women in the second trimester: 30.4 ± 21.6 MET-h/week, group of women in the third trimester: 35.5 ± 23.1 MET-h/week) and *moderate activity* (42.7 ± 45.2 and 39.4 ± 52.8 MET-h/week, respectively).

Analysis of the type of activities showed that the MET-h/week values did not differ between the groups studied for *household*/*caregiving*, *occupational activity*, *transportation* and *sports*/*exercise*. However, it was noticeable that higher energy expenditure (*p* ≤ 0.01) in women in the third trimester of pregnancy was observed for the activities related to *inactivity* (51.4 ± 28.87 MET-h/week) compared to those in the second trimester (42.7 ± 24.9 MET-h/week).

The WHOQoL-Bref results indicated no differences in the self-rated quality of life of the women surveyed (Table 2). Pregnant women in both the second and third trimesters rated their quality of life in the *psychological domain* as the highest (16.48 ± 1.88 in the second trimester and 16.56 ± 1.64 in the third trimester), whereas the lowest ratings were recorded for the *environmental domain* (15.89 ± 1.96 and 15.78 ± 1.91, respectively).

The next stage of the statistical analysis focused on investigating whether there is a correlation between intensity and types of PA assessed using PPAQ-PL and domains of the QoL assessed using WHOQoL-Bref in women in the second (Table 3) and third (Table 4) trimesters of pregnancy.

There was a significant correlation in the group of women in the second trimester of pregnancy for *physical health domain* with intensity and the type of activities (Table 3). The women who rated their quality of life higher in this domain declared higher energy expenditures associated with *vigorous activity* (*R* = 0.159, *p* ≤ 0.05), as well as with *occupational activity* (*R* = 0.166; *p* ≤ 0.05) and *sport*/*exercise* activity (*R* = 0.187; *p* ≤ 0.05).

Furthermore, a negative correlation was found between *physical health domain* and *inactivity* (*R* = −0.151, *p* ≤ 0.05). This means that higher assessment of quality of life in this domain coincided with lower energy expenditure related to *inactivity*. Individual positive correlations were also documented between *social relationship domain* and *transportation activity* (*R* = 0.166, *p* ≤ 0.05) and between *environmental domain* and *sport*/*exercise activity* (*R* = 0.103, *p* ≤ 0.05).

In women in the third trimester, higher energy expenditures related to *sport*/*exercise activity* coincided with higher assessments of the *overall quality of life* (*R* = 0.149, *p* ≤ 0.05) and *general health* (*R* = 0.170, *p* ≤ 0.05). In the case of the *psychological domain* (*R* = 0.161, *p* ≤ 0.05) and *social relationship domain* (*R* = 0.188; *p* ≤ 0.05) of QoL, positive correlations occurred with energy expenditure related to *vigorous activity*. In contrast, high assessment of the *physical health domain* coincided with higher energy expenditure related to *occupational activity* (*R* = 0.174; *p* ≤ 0.05).

## 4. Discussion

The aim of this study was to investigate relationships between PA and QoL in women in the second and third trimesters of pregnancy. The respondents’ declarations from the PPAQ-PL and WHOQoL-Bref questionnaires were analyzed. Although studies have used these questionnaires in recent years [16,18], this is, to our knowledge, the first such study in Poland. Such research is also important because previous findings concerning PA in women in various stages of pregnancy have been ambiguous. Some authors have found increases in PA, for example, Huberty et al. [10] in the first and second trimesters, and Ko, Chen, Lin [38] from the second trimester, while others have documented a decline in overall PA [38] in the first trimester, Evenson and Wen [39] in the third trimester, and a decreasing percentage of physically active women in consecutive trimesters of pregnancy [2]. Borodulin et al. [8] argued that the overall physical activity level slightly decreased between 17–22 and 27–30 weeks of gestation, particularly in duration and volume of care, outdoor household, and recreational activity. Santos et al. [40] emphasized that a decline in PA from the first to the second trimester concerned total, light and moderate intensities, while Richardsen et al. [41], documented a decline in moderate and vigorous PA in the period between early pregnancy and mid-pregnancy.

Similar to findings published by Mourady et al. [16], our findings showed that the respondents in different trimesters of pregnancy did not differ in terms of *total PA*, and *total activity of light intensity and above*. However, they differed in the intensity of activities, especially in *energy expenditure* during *moderate activity* (in favor of those in the second trimester). The women in the third trimester of pregnancy reported significantly more energy expenditure on *sedentary activity* and *inactivity*, which is not an isolated phenomenon in the world [10]. According to Santos et al. [40], energy expenditure for particular types of PA (e.g., occupational activity, household activity, sports activity) changes significantly in individual trimesters of pregnancy. Pregnant women spent most of their weekly time on domestic, occupational and leisure time activities, except for sports activities. Similarly, the majority of the respondents’ energy expenditure in the respondents surveyed in our study was spent on *household*/*caregiving activities*. This suggests that despite many campaigns to raise awareness of Polish women, such as: Pregnancy: Conscious Maternity, Find Out Whether You Are a Conscious Parent, or Different State, Different Treatment (Ciąża-Świadome Macierzyństwo, Sprawdź czy jesteś świadomym rodzicem, Odmienny stan, odmienne traktowanie), the role of the benefits of PA during pregnancy remains underestimated. In the public’s opinion, healthy nutrition is more often perceived as more important for the health of mothers and children than involvement in physical activity [42]. Therefore, the low levels of energy expenditure related to *sport*/*exercise activity* found in the present study in both groups of women surveyed seem unsurprising. Perhaps, as argued by Clarke and Gross [11] and Guelfi et al. [43], women perceive relaxation as a safer behavior, which is more beneficial for ensuring full-term pregnancy rather than regular exercise and maintaining an active lifestyle. The reasons also include misconceptions about physical exercise [44], the inconveniences of late pregnancy, fatigue, poor moods, or being absorbed in numerous occupational duties [45,46]. There are also other determinants that represent barriers to physical exercise. For example, low physical activity during pregnancy occurs more often in mature and married women, as well as those financially less well-off and the less educated [47]. It seems, however, that regardless of the adversities, the role of physicians is also critical as they have the greatest effect on the beliefs of pregnant women, including their ideas on exercise during pregnancy [48]. Unfortunately, as Santos et al. [40] argued, medical staff often fail to recommend PA during pregnancy. Furthermore, according to Krans et al. [48], a low percentage of physicians help their patients to prepare physical exercise programs. Despite their knowledge, physicians do not always explain the need for physical exercise, both during pregnancy and in later decades of life [49]. They do not inform patients that it is necessary to consult both physicians and coaches before starting physical exercise in order to exclude medical contraindications and choose the right type of exercises and the load.

Knowledge about the quality of life plays a significant role both in diagnosis and patient care [50]. Despite being ambiguous, studies have widely documented the correlations between physical activity and quality of life. According to the literature review published by Poudevigne et al. [47], there is scientific evidence that inactivity during pregnancy is associated with poorer mood, whereas increasing participation in sports or physical activity from the period of pregnancy to that after birth leads to better overall well-being [51]. Mourady et al. [16] demonstrated that total and light intensity of PA are positively significantly correlated with the psychological domain of quality of life and social relationships; while sedentary PA is significantly correlated only with social relationships. Arizabaleta et al. [19] documented improvements in HRQoL in the physical component summary, physical function domain, the bodily pain domain and general health domain following a three-month program of aerobic exercise. However, there are also publications that showed no improvements in self-rated QoL caused by regular exercise such as water exercise [22].

Analysis of QoL of the women surveyed showed that there were no significant differences in self-rated domains of WHOOoL-Bref between pregnant women in the second and third trimesters of pregnancy. Similarly, Mourady et al. [16], who analyzed all the trimesters, also found no differences except in the environmental domain. In this case, the quality of life was significantly higher in the women in the third trimester compared to those in the first trimester.

In our study, we found higher QoL scores in the *environment domain* in women in the second trimester who declared higher *sport*/*exercise activity.* It should also be noted that *sport*/*exercise activity* of the respondents studied was also positively correlated with the *physical domain* in women in the second trimester and with *overall quality of life* and *general health* in women in the third.

Our findings are consistent with those presented by Mourady et al. [16], who showed that sports/exercise was significantly correlated with the majority of quality of life domains such as general quality of life, physical and psychological health, social relationships and the environmental domain. This is unsurprising since apart from its well-documented health benefits, sport [5,6,7] offers joy, relaxation and enhances psychological well-being [52]. Obviously, there have also been studies in the literature that have failed to support such findings. For example, Gustafsson et al. [53] indicated that a 12-week exercise program including aerobic and strength training during pregnancy is unlikely to influence the psychological and self-perceived well-being of healthy pregnant women. Kolu et al. [17] showed a decline in the overall HRQoL index during pregnancy, although they emphasized that this decrease was lower in women who were physically active during pregnancy. Nascimento et al. [23] argued that physical exercise does not significantly affect the perception of the quality of life of pregnant women because, regardless of their participation in the exercise program, the quality of life of women (in the physical and social domains) during pregnancy fell significantly.

An interesting finding of our study is that pregnant women who assessed QoL as higher in the field of *physical health* (both in the second and third trimester) were characterized by a higher energy expenditure during *occupational activity*. The explanation for this finding seems to be obvious; women with better self-rated physical well-being tend to work more. We are aware, however, that the explanation for this phenomenon may be more complex. The study published by Blum et al. [51] showed that women with older infants or no other children reported higher household/caregiving and lower occupation pre-pregnancy to postpartum activity. Physical activity in pregnancy may depend on the socio-economic status and support of a partner, friends or family [54,55]. These factors may, to a large extent, determine the quality of life [56]. Unfortunately, due to the lack of the above-mentioned information in our study, the impact of these factors was impossible to determine. Some limitations of this study should be mentioned and taken into consideration. First of all, a limited number of participants and the place where the women were selected (fitness clubs and antenatal classes) lead to a lack of representativeness of the total population with possible effects on the results. This in turn makes it impossible to draw general conclusions for the whole population of pregnant women. Furthermore, the lack of detailed information on socio-economic and psychological factors and data about pathologies makes the interpretation of the results difficult. 

## 5. Conclusions

In conclusion, our study (the first study in Poland that has used reliable, internationally recognized questionnaires (PPAQ-PL and WHOQoL-Bref) makes an important contribution to the knowledge concerning the correlations between PA and QoL in women during different periods of pregnancy. The study showed that *total activity* and *total activity of light intensity and above* did not differentiate between women in the second and third trimesters of pregnancy. However, it indicated higher values of *moderate activity* in women in the second trimester of pregnancy and higher values of *sedentary activity* and *inactivity* in women in the third trimester. Our findings concerning the relationships between physical activity and quality of life should be approached with caution, due to the low values of correlation coefficients. The low MET values for sport/exercise recorded in both groups of women can indicate the need for improving the prenatal care, especially in terms of promotion of physical activity programs for pregnant women and encouraging women to participate in these programs. In terms of intervention activities, special attention should be given to barriers existing at the level of the provider, the patient, and practice [57]. Researchers [58] have specified concrete actions that should be taken by prenatal care providers in order to promote prenatal PA, suggesting, among other things, providing information by healthcare providers about both guidelines and contraindications for the involvement in physical activity during pregnancy. Most guidelines from around the world, gathered by Evenson et al. [59], promoted moderate-intensity physical activity during pregnancy and defined its frequency and duration/time. The latest physical activity guidelines published by the U.S. Department of Health and Human Services USDHHS in 2018 [60], indicate 150 min (2 h and 30 min) of moderate-intensity aerobic activity a week during pregnancy and the postpartum period. Recommending aerobic activity should be spread throughout the week.

Undoubtedly, our study does not exhaust the problems discussed but its findings emphasize the need for raising awareness of the importance of physical activity during pregnancy. We believe that further research on a larger sample with the consideration of socio-economic factors and a comprehensive inventory of pregnancy-related symptoms, along with a mechanism for assessing their effect on function [61] is needed to provide deeper understanding and identify correlations between PA and determinants of QoL in pregnant women.

## Figures and Tables

**Table 1:** Means (M), standard deviations (SD), medians, and 25th and 75th percentiles for the Pregnancy Physical Activity Questionnaire (PPAQ-PL) and intergroup comparisons (the second and third trimesters) using the Mann–Whitney U-test.

Factors	Trimester	PPAQ-PL (MET-h/week)				
		M ± SD	25th	Median	75th	*p*
**Total Activity Scores:**						
Total activity	2nd	183.3 ± 75.2	129.1	166.8	220.7	0.721
	3rd	192.1 ± 99.3	130.4	168.8	227.2	
Total activity of light intensity and above	2nd	152.9 ± 75.9	99.7	143.3	184.2	0.838
	3rd	156.6 ± 95.5	99.2	136.9	188.7	
**by Intensity:**						
Sedentary (<1.5 METs)	2nd	30.4 ± 21.6	15.4	29.4	43.4	0.025 *
	3rd	35.5 ± 23.1	17.9	29.4	46.2	
Light (1.5–<3.0 METs)	2nd	110.4 ± 547.4	73.3	104.7	140.7	0.355
	3rd	117.8 ± 55.3	73.5	109.8	151.6	
Moderate (3.0–6.0 METs)	2nd	42.7 ± 45.2	17.9	30.9	50.2	0.022 *
	3rd	39.4 ± 52.8	13.7	23.7	46.9	
Vigorous (>6.0 METs)	2nd	1.78 ± 4.2	0.0	0.0	0.8	0.937
	3rd	1.6 ± 4.3	0.0	0.0	0.8	
**by Type:**						
Household/Caregiving	2nd	56.4 ± 39.8	33.6	43.5	67.6	0.316
	3rd	59.1 ± 40.1	33.9	50.1	70.4	
Occupational activity	2nd	37.8 ± 60.9	0.00	0.00	74.9	0.337
	3rd	33.2 ± 61.4	0.0	0.0	67.2	
Sports/Exercise	2nd	12.8 ± 11.8	7.2	12.8	21.2	0.212
	3rd	14.5 ± 12.7	5.0	11.0	19.7	
Transportation	2nd	30.9 ± 31.1	10.7	21.4	36.8	0.505
	3rd	33.84 ± 34.5	10.7	22.6	42.0	
Inactivity	2nd	42.7 ± 24.9	24.2	38.2	57.4	0.005 **
	3rd	51.4 ± 28.87	29.6	44.9	70.0	

MET-Metabolic Equivalent of Task; * *p* ≤ 0.05; ** *p* ≤ 0.01.

**Table 2:** Medians, 25th and 75th percentiles, means (M) and standard deviations (SD) for the Quality of Life-Bref Questionnaire (WHOQoL-Bref) and intergroup comparisons (2nd and 3rd trimesters) using the Mann–Whitney U-test.

WHOQoL-Bref Factors	Trimester	M ± SD	25th	Median	75th	p
Overall quality of life	2nd	4.34 ± 0.61	4.00	4.00	5.00	0.300
	3rd	4.41 ± 0.65	4.00	4.00	5.00	
General health	2nd	4.13 ± 0.67	4.00	5.00	5.00	0.507
	3rd	4.19 ± 0.65	4.00	4.00	5.00	
**WHO Domain**						
Physical health	2nd	16.06 ± 2.16	15.00	16.00	18.00	0.187
	3rd	15.80 ± 01.99	14.00	16.00	17.00	
Psychological	2nd	16.48 ± 1.88	15.00	17.00	17.00	0.552
	3rd	16.56 ± 1.64	15.00	17.00	17.00	
Social relationships	2nd	16.47 ± 2.21	16.00	16.00	19.00	0.274
	3rd	16.14 ± 2.60	15.00	16.00	17.00	
Environmental	2nd	15.89 ± 1.96	15.00	16.00	17.00	0.781
	3rd	15.78 ± 1.91	15.00	16.00	17.00	

**Table 3:** Spearman’s correlation coefficients between the Pregnancy Physical Activity questionnaire (PPAQ-PL) and Quality of Life-Bref Questionnaire (WHOQoL-Bref) in women in the second trimester of pregnancy.

	WHOQoL-Bref	Overall Quality of Life	General Health	WHOQoL-Bref Domain			
PPAQ-PL				Physical Health	psychological	Social Relationships	Environmental
**Total Activity Scores:**							
Total activity		−0.003	0.005	0.057	0.020	0.071	−0.061
Total activity of light intensity and above		0.002	0.007	0.089	0.037	0.129	−0.037
**by Intensity**							
Sedentary (<1.5 METs)		−0.040	−0.028	−0.041	−0.072	−0.104	−0.073
Light (1.5–<3.0 METs)		0.029	−0.025	0.129	0.061	0.091	0.011
Moderate (3.0–6.0 METs)		−0.035	0.043	0.029	−0.003	0.128	−0.073
Vigorous (>6.0 METs)		−0.065	0.043	0.159 *	0.072	0.122	0.079
**by Type**							
Household/Caregiving		0.0125	−0.059	−0.116	−0.143	−0.008	−0.035
Occupational activity		0.003	0.047	0.166 *	0.117	0.105	0.040
Sports/Exercise		0.035	0.081	0.187 *	0.103	0.153	0.103 *
Transportation		0.020	0.053	0.111	0.155	0.166 *	0.054
Inactivity		0.022	−0.017	−0.151 *	−0.075	−0.097	−0.004

MET-Metabolic Equivalent of Task; * *p* ≤ 0.05.

**Table 4:** Spearman’s correlation coefficients between the Pregnancy Physical Activity questionnaire (PPAQ-PL) and Quality of Life-Bref Questionnaire (WHOQoL-Bref) in women in the third trimester of pregnancy.

	WHOQoL-Bref	Overall Quality of Life	General Health	WHOQoL-Bref Domain			
PPAQ-PL				Physical Health	Psychological	Social Relationships	Environmental
Total activity		−0.068	0.052	0.048	0.003	0.036	−0.094
Total activity of light intensity and above		−0.049	0.049	0.085	−0.029	−0.009	−0.073
Sedentary (<1.5 METs)		−0.090	−0.018	−0.125	0.020	0.0463	−0.068
Light (1.5–<3.0 METs)		−0.053	−0.013	0.065	−0.068	−0.019	−0.133
Moderate (3.0–6.0 METs)		0.010	0.140	0.117	0.054	0.040	0.099
Vigorous (>6.0 METs)		0.029	0.041	0.073	0.161 *	0.188 *	−0.072
Household/Caregiving		−0.112	0.056	−0.023	0.023	−0.037	−0.059
Occupational activity		0.050	0.038	0.174 *	−0.024	−0.013	0.034
Sports/Exercise		0.149 *	0.170 *	0.101	0.087	0.067	0.131
Transportation		−0.064	−0.0083	0.068	−0.060	0.033	−0.124
Inactivity		−0.077	−0.034	−0.128	−0.028	0.028	−0.096

MET-Metabolic Equivalent of Task; * *p* ≤ 0.05.
